# Failure Mechanism of Pre-Stressed CFRP Beam Under Laser Ablation

**DOI:** 10.3390/polym17152153

**Published:** 2025-08-06

**Authors:** Yuting Zhao, Ruokun Zhang, Zhuhua Tan

**Affiliations:** School of Mechanical Engineering, Hebei University of Technology, Tianjin 300400, China; 18531690200@163.com (Y.Z.); rkzhang0512@163.com (R.Z.)

**Keywords:** CFRP beam, laser ablation, failure mechanism, pyrolysis, delamination

## Abstract

This paper focuses on the failure mechanism of a pre-stressed CFRP cantilever beam under laser ablation. During testing, a mass was applied to the CFRP cantilever beam to achieve a pre-stressed state, and the laser power densities varied from 500 to 1500 W·cm^−2^. Corresponding scanning electron microscope (SEM) tests were also performed on the ablation zone and fracture surface to analyze the failure mechanism. The results showed that the CFRP beam failed in compression at the bottom surface, which was due to a decrease in local stiffness and strength caused by heat softening, rather than by ablation damage on the top surface. The failure time decreased from 19.64 s to 6.52 s as the power density (500–1500 W·cm^−2^) and pre-stress loading (300–750 N·cm) increased, indicating that pre-stress loading has a more significant influence on the failure time of CFRP beams compared to power density.

## 1. Introduction

Carbon fiber-reinforced polymer (CFRP) materials have been widely applied in the fields of aerospace, transport, and building structures due to their excellent mechanical and physical properties, such as a high specific strength, high specific stiffness, and excellent corrosion resistance [1]. Extensive work has been performed to investigate the mechanical response of CFRP materials and structures (sandwich structures [2,3], lattice structures [4,5], tubes [6]) subjected to quasi- and dynamic loads, focusing on the deformation and failure mechanisms of delamination [7], fracture [8], and collapse [9]. During service life, CFRP materials and structures are also subjected to fire/heat loading, which threatens the safety and reliability of CFRP materials and structures. Compared to research on the responses of CFRP materials under mechanical loading, studies involving high-temperature/heat flux loading are rare. Moreover, the mechanical properties of CFRP materials are sensitive to temperature. So, it is necessary to study the influence of high heat flux loading on the mechanical performance of CFRP materials and structures.

Generally, CFRP materials contain two categories based on their matrix: thermoset CFRP (matrix made of epoxy resin, etc.) and thermoplastic CFRP (matrix made of nylon, copolymerized acrylic, polycarbonate, etc.) [10,11]. The thermoplastic matrix can improve fracture toughness, flexural strength, and the flexural modulus. Thermoset CFRP materials have excellent mechanical properties (tensile strength, hardness, peak load, yield strength, etc.), which makes them attractive for prospective applications. It is well known that the matrix of thermoset CFRP materials changes from a glass state to a rubber state at a specific temperature, which results in a serious decrease in the stiffness and strength of the CFRP by 85% and 65%, respectively [12,13]. Moreover, when the temperature increases further (>300 °C), the polymer matrix of CFRP starts to pyrolyze into char, CO_2_, H_2_O, etc. However, the property evolution law and mechanisms of carbon fiber composites at high temperatures are not sufficient [14]. Thus, the response of CFRP materials and structures under high heat flux loading is a complicated thermal–mechanical–chemical coupling problem, which has also attracted significant attention and interest.

Recently, studies have been conducted on the performance of CFRP materials at high temperatures, such as thermal conduction [15,16,17,18,19,20,21], pyrolysis [22,23,24,25,26,27,28,29,30,31,32], and ablation charring [24,33,34,35,36,37,38,39]. For example, Koyanagi et al. [26] studied the delamination of mid-density CFRP caused by internal gas pressure under rapid heating. Kandare et al. [27] investigated the thermal–mechanical response of fiber-reinforced composites subjected to heat fluxes of 2.5–3.5 W·cm^−2^ experimentally and numerically. Chippendale et al. [28] developed a numerical model to study the decomposition of CFRP. Li et al. [35] established a thermal–mechanical–chemical model to evaluate the ablation morphology for high-energy laser ablation of CFRP. Most of these works focused on CFRP materials and the corresponding ablation and pyrolysis mechanisms, which paved the way for investigating the responses of CFRP structures subjected to heating–mechanical loading.

In the present paper, high-energy laser ablation on a pre-stressed CFRP beam was performed. During the tests, different laser power densities (500, 1000, and 1500 W·cm^−2^) and pre-stress loadings (300, 500, and 750 N·cm) were applied on a CFRP cantilever beam, and the displacement of the CFRP beam was also recorded by a laser distance meter. Based on the experimental and SEM test results, the influence of the laser power density and pre-stress loading on the mechanical response was analyzed. The deformation and failure mechanism of the pre-stressed CFRP cantilever beam were also discussed.

## 2. Experiments

### 2.1. CFRP Materials and Specimens

**Figure 1:**
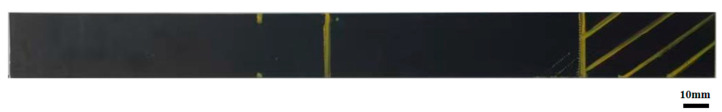
Graph of the CFRP beam specimen.

The CFRP composite was laminated by 32 plies of T800 unidirectional carbon fiber/epoxy resin prepreg (produced by the Changzhou Teruo composite Company in Changzhou, China). For the carbon fiber-reinforced epoxy resin prepreg materials, the area density was about 194 ± 5 g/m^2^, the elastic modulus was about 155 GPa, the tensile strength in the $0^{{}^{\circ}}$ direction was about 2800 MPa, and the compressive strength in the $0^{{}^{\circ}}$ direction was about 1500 MPa. The plies were laid in accordance with an orthogonal sequence of [0°, 90°] s, and the thickness of each layer was 0.125 mm. The specimens of the CFRP beam were cut from a T800/epoxy resin CFRP panel. The dimensions of the CFRP beam measured 200 mm in length × 30 mm in width × 2 mm in thickness, as shown in Figure 1.

### 2.2. Experimental Apparatus

**Figure 2:**
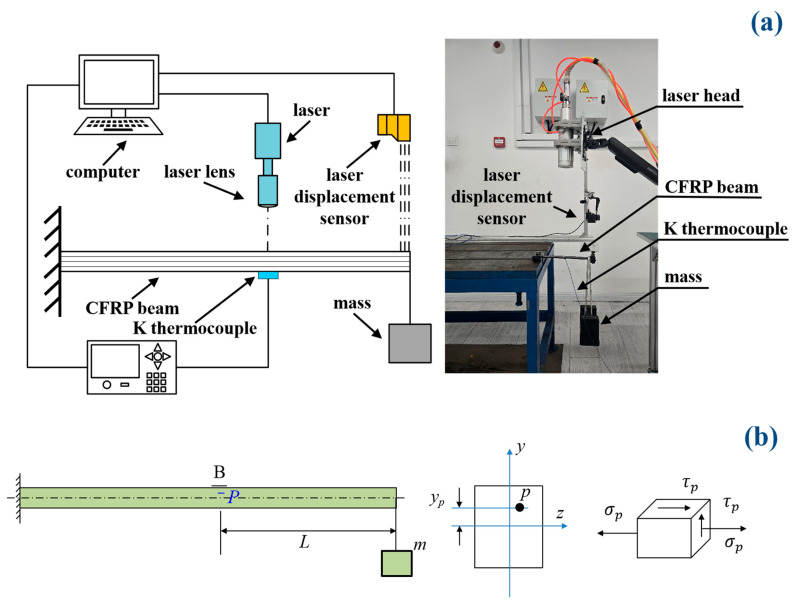
Schematic of the experimental apparatus and pre-stress state. (**a**) Experimental apparatus; (**b**) pre-stress state.

Figure 2a illustrates the schematic of the laser ablation experiment. The laser ablation test of the pre-stressed CFRP cantilever beam was conducted using a Raycus CS6000 CW laser (product of Raycus Company, Wuhan, China). The parameters of the CW fiber laser device are as follows: the wavelength of the continuous wave laser is 1080 ± 5 nm, the spot diameter is 20 mm, the Rayleigh length is 1.680 mm, the value of the $M^{2}$ is 8.31, and the corresponding beam parameter product (BBP) is about 2.856 mm·mrad.

During the tests, the pre-stress of the CFRP cantilever beam was achieved by applying a 2 kg and 5 kg mass on the free ends of the beam, respectively, as shown in Figure 2a. Subsequently, specific CW power levels (P) of the laser device were set as 1570 W, 3140 W, and 4710 W, respectively. The corresponding power density could then be calculated by a ratio of the laser power to the spot area $\left(P/A=\frac{4P}{\pi D^{2}}\right)$. Thus, laser power densities of 500 W·cm^−2^, 1000 W·cm^−2^, and 1500 W·cm^−2^ were applied on the different positions of the CFRP beam, respectively. A K-type thermocouple was used to measure the temperature, and the measurement point was located beneath the laser spot center on the bottom surface of the CFRP beam. A maximum temperature was recorded to analyze the thermal softening efficiency. The load cases with different masses and power densities were designed and are listed in Table 1.

The pre-stress state was induced by the applied mass (*m*), which included not only a normal stress $\sigma_{p}$ but also a shear stress $\tau_{p}$, as shown in Figure 2b. These pre-stresses can be calculated based on the theory of the stress state in a beam; the details are as follows:

(1) $$\sigma_{p}=\frac{My_{p}}{EI_{z}}=\frac{(mgL)y_{p}}{EI_{z}}$$

(2) $$\tau_{p}=\frac{F_{s}}{2I_{z}}(\frac{h^{2}}{4}-y_{p}^{2})=\frac{mg}{2I_{z}}(\frac{h^{2}}{4}-y_{p}^{2})$$

where $M\;and\;F_{s}$ are the moment and shear force induced by the applied mass, respectively; $y_{p}$ is the distance between point P and the neutral axis of *z*; E is the effective elastic modulus of the CFRP; $I_{z}$ is the inertia moment of the cross-section; h is the height of the beam; m is the applied mass; and L is the distance between the free end and the laser’s position. To achieve the different pre-stresses, we can adjust the parameters of m and L. In order to distinguish the pre-stress state and pre-stress loading, the pre-stress loading was marked by the moment (M=mgL), and the pre-stress state was marked by normal stress $\left(\sigma_{p}\right)$ and shear stress ${(\tau }_{p})$, as shown in Formulas (1) and (2). For example, if the mass is 5 kg and the distance is 10 cm, then the pre-stress loading is 500 N·cm.

To analyze the failure mechanism of the pre-stressed CFRP beam, the displacement of the free end of the CFRP cantilever beam was monitored by a laser optical displacement sensor, and the sampling rate of the laser distance meter was 10 Hz.

### 2.3. SEM and TG Tests

After the ablation experiments, the ablation and failure surfaces of the CFRP beam were observed using a Hitachi S-4700 scanning electron microscope (SEM). The SEM tests were performed to characterize the details of the ablation, pyrolysis, and failure. Moreover, the thermogravimetric (TG) analysis was also carried out by a Mettler TGA/DSC 3+ apparatus. The 1.5 g CFRP sample was tested in an aluminum crucible, and heated from 20 °C to 1000 °C in a nitrogen gas atmosphere at a heating rate of 10 °C/min.

## 3. Results and Discussions

### 3.1. Failure Modes and Failure Time

**Figure 3:**
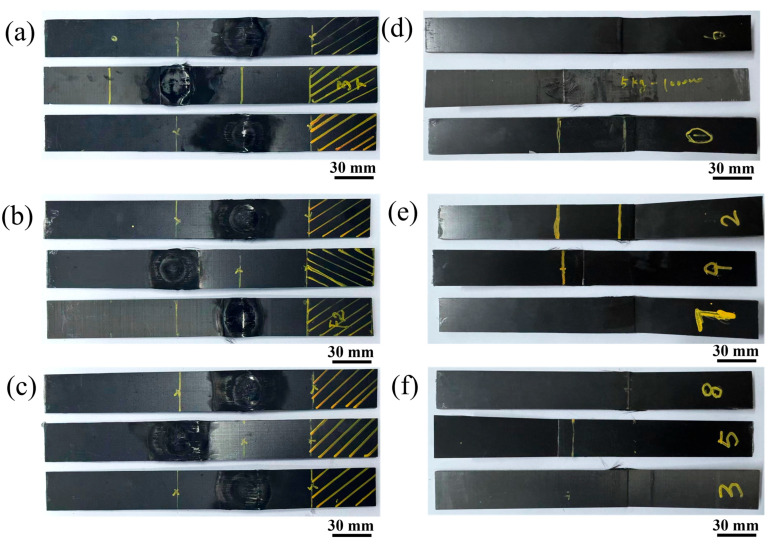
The failure modes of the CFRP beam under different power densities. (**a**,**d**), (**b**,**e**), and (**c**,**f**) show the top surfaces and bottom surfaces of the CFRP beam under 500 W/cm^2^, 1000 W/cm^2^, and 1500 W/cm^2^, respectively.

Figure 3 shows the ablation damage and failure modes of the CFRP cantilever beam under different load cases. Although the power densities and pre-stress states were different, the ablation damage and failure modes were similar to each other. However, severe ablation damage occurred on the top surface of the CFRP beam, the carbon fibers were exposed, and the corresponding epoxy resin ablated and pyrolyzed completely, as shown in Figure 3a–c. Although the carbon fibers were exposed, they were still intact.

Unlike the ablation damage on the top surface of the CFRP beam, there was an obvious fracture failure mode on the bottom surface, as shown in Figure 3d–f. It is well known that, for a cantilever beam, tensile stress develops on the top surface and compressive stress on the bottom surface. Thus, the failure modes in Figure 3d–f are the compressive failure modes. It can be seen that the CFRP cantilever beam failed in compression failure mode on the bottom surface, which provided strong evidence for analyzing the failure mechanism of the pre-stressed CFRP beam subjected to laser ablation. Moreover, significant delamination was observed in the tested specimens, which may have been caused by thermal expansion and internal gas induced by pyrolysis [26,27].

**Figure 4:**
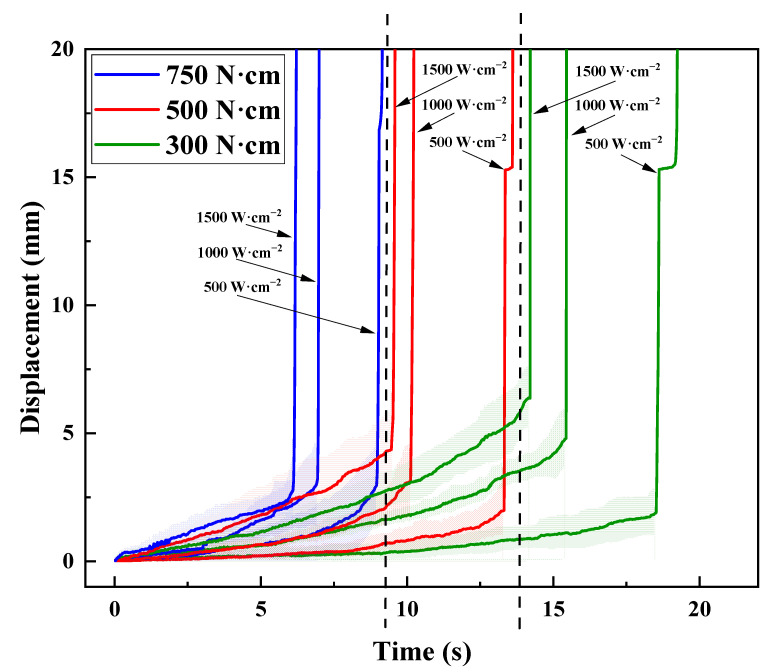
The displacement-versus-time curves of the free ends of the pre-stressed CFRP beam under different power densities.

Figure 4 illustrates the curves of the displacement versus time of the free ends of the CFRP cantilever beam. The shaded area represents the standard deviation of the results. The error is small, indicating that the thermal–mechanical response was well captured during the tests. It is also clear that these curves are distributed across three regions, with each exhibiting an obvious influence by the pre-stress loading. Generally, the failure time of the pre-stress loading case of 300 N·cm is nearly 2 times of that of 750 N·cm. In Figure 4, each displacement–time curve has a similar variation tendency. Generally, each curve contains two stages. Firstly, the displacement increases with time, which means that the CFRP beam deforms and still bears the thermal–mechanical couple loading. Subsequently, a turn out point is observed, and then a steep increase follows, indicating that the CFRP beam loses its load-bearing capability and fails. Similar variation tendencies were also reported in Refs. [40,41]. Tension stress develops on the top surface of the CFRP cantilever beam, and the exposed carbon fibers can still bear the tensile stress, which corresponds to the ablation damage, as shown in Figure 3a–c. Sauder et al. [42] reported that the tensile strength of different carbon fibers nearly does not decrease when the temperature is below 1600 °C, and that there is about a 20% decrease in tensile strength as the temperature rises up to 2000 °C. This means that laser ablation has almost no influence on the load bearing in the tension of the CFRP beam. Furthermore, a sudden jump can be seen in the curves, which indicates the collapse of the CFRP cantilever beam, corresponding to the compressive failure modes on the bottom surface, as shown in Figure 3d–f. Moreover, for a specific power density/pre-stress loading, the failure time decreased with the increase in the pre-stress loading/power density, respectively. This means that both power density and pre-stress loading have an obvious influence on the failure time.

In order to analyze the influence of the power density and pre-stress loading on the failure time, a two-way ANOVA method was used to evaluate the contribution of the power density and pre-stress loading to the failure time, and the corresponding results are listed in Table 2. All the values of *p*-value in Table 2 are less than 0.05, which indicates that both power density and pre-stress loading have an obvious influence on the failure time. Moreover, the contributions of the power density and pre-stress loading are 16.7% and 79.8%, respectively, and their coupling contribution is 2.1%. The results listed in Table 2 show that the pre-stress loading has a greater influence compared to power density.

**Figure 5:**
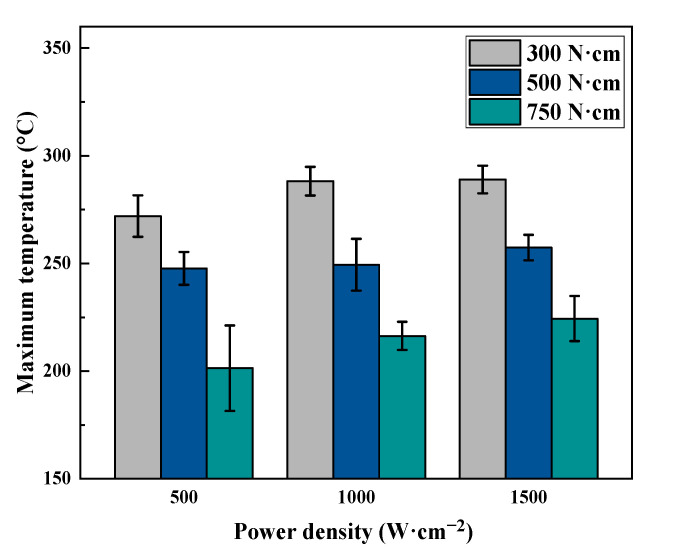
The maximum temperature of the position beneath the laser spot center on the bottom surface of the CFRP beam.

Moreover, similarly to Figure 1, the maximum temperature of the position beneath the laser spot center on the bottom surface of the CFRP beam was recorded and is shown in Figure 5. Generally, the maximum temperature decreases with the increase in pre-stress loading for each power density case. And the maximum temperatures on the bottom surface of the CFRP beam under 500 W·cm^−2^, 1000 W·cm^−2^, and 1500 W·cm^−2^ are approximately 280 °C, 250 °C, and 210 °C for the pre-stress loadings of 300 N·cm, 500 N·cm, and 750 N·cm, respectively. This variation tendency agrees well with the variation in failure time. When the power density is 500 W·cm^−2^ and pre-stress loading is 300 N·cm, the failure time is about 19.64 s; however, for a power density of 500 W·cm^−2^ and pre-stress loading of 750 N·cm, the failure time is just 9.44 s. So, less time equates to less heat transferring. The corresponding maximum temperature is also low.

### 3.2. Influence of Power Density and Pre-Loadings on Failure Time

**Figure 6:**
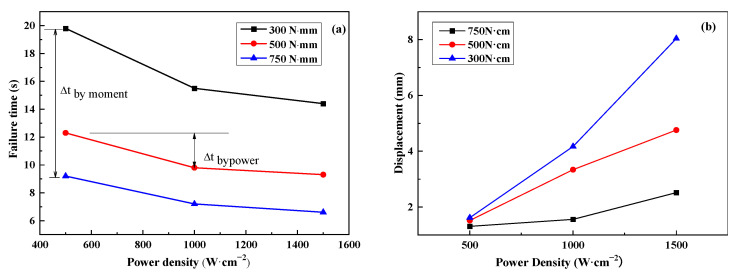
The influence of power density and pre-stress loading on failure time and displacement: (**a**) failure time; (**b**) displacement.

Figure 6a illustrates the variation in failure time with laser power density under different pre-moment loads. It can be observed that the pre-moment load has a greater influence on the failure time than laser power density. There is an obvious decrease in failure time when the pre-moment load is increased. For example, for a specific power density of 500 W·cm^−2^, when the pre-moment load increases from 300 to 750 N·cm, the failure time decreases from 19.72 s to 9.2 s. Compared to 300 N·cm, the variation in the failure time by the pre-moment Δt_bymoment_ is about 7.5 s for 500 N·cm, and 10.52 s for 750 N·cm. A similar variation tendency in failure time can also be found for laser power values of 1000 W/cm^2^ (Δt_bymoment_ is 5.7 s and 8.3 s) and 1500 W·cm^−2^ (Δt_bymoment_ is 5.1 s and 7.8 s).

However, under a specific pre-moment load of 300 N·cm, as the power density increases from 500 to 1500 W/cm^2^, the failure time decreases from 19.76 s to 14.4 s. This variation in failure time by power density Δt_bypower_ is about 4.26 s at 1000 W·cm^−2^ and 5.36 s at 1500 W·cm^−2^ compared to 500 W·cm^−2^, respectively, which is much less than Δt_bymoment_. A similar variation tendency in failure time can also be found for pre-moment loads of 500 N·cm (Δt_bypower_ is 2.5 s and 3.1 s) and 750 N·cm (Δt_bypower_ is 2.0 s and 2.6 s).

Figure 6b illustrates the influence of the power density and pre-stress loading on the displacement of the free ends of the CFRP beam. During the laser irradiation, for a given pre-stress loading, the displacement of the free end of the CFRP beam increased with the increase in the power density. The higher the power density, the larger the variation tendency. When the pre-stress loading was 300 N·cm, the displacements were 1.62 mm, 4.17 mm, and 8.04 mm under power densities of 500 W·cm^−2^, 1000 W·cm^−2^, and 1500 W·cm^−2^, respectively. The maximum variation was 6.42 mm. As the pre-stress loading increased to 750 N·cm, the displacements were 1.31 mm, 1.56 mm, and 2.52 mm under power densities of 500 W·cm^−2^, 1000 W·cm^−2^, and 1500 W·cm^−2^, respectively. The corresponding maximum variation was 1.21 mm, which is about 1/5 of the pre-stress loading of 300 N·cm. This means that power density has an obvious contribution to the displacement; however, the influence decreases with increasing pre-stress loading.

On the contrary, for a given power density, the displacement of the free ends of the CFRP beam decreased with the increase in pre-stress loading. And the variation magnitude increased with increasing pre-stress loading. For the case of 500 W·cm^−2^, the displacements were 1.62 mm, 1.52 mm, and 1.31 mm under pre-stress loadings of 300 N·cm, 500 N·cm, and 750 N·cm, respectively. The maximum difference was just 0.31 mm. However, for the case of 1500 W·cm^−2^, the displacements were just 8.04 mm, 4.76 mm, and 2.52 mm under pre-stress loadings of 300 N·cm, 500 N·cm, and 750 N·cm, respectively. The maximum difference was about 5.52 mm, which is about 17 times that of the case of 500 W·cm^−2^. But this value of 5.52 mm obtained under different pre-stress loadings is still lower than the 6.42 mm obtained by different power densities. Thus, compared to the pre-stress loading, power density has a greater impact on the displacement of the CFRP beam.

### 3.3. Deformation and Failure Mechanism

**Figure 7:**
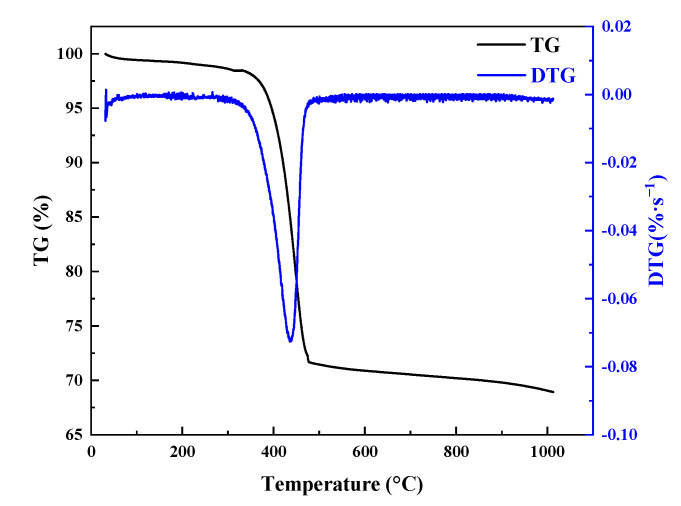
The curves of the TG and DTG results.

Figure 7 presents the TG and DTG curves of the CFRP materials. There are three stages of the TG/DTG curves. Firstly, a minor weight loss (less than 3%) can be observed below 100 °C, which is caused by the evaporation of the water. Subsequently, significant weight loss occurs within the range of 375 to 455 °C. Finally, the weight loss is approximately zero when the temperature is higher than 455 °C. Moreover, the material exhibits a significant weight loss peak near 415 °C, which is due to the pyrolysis of epoxy resin. Additionally, it is clear that the glass transition started at a temperature of ~375 °C, while the pyrolysis ended at a temperature of ~455 °C, which agree with the results of Ref. [11]. The residual pyrolysis products are about 70% of the original CFRP materials.

**Figure 8:**
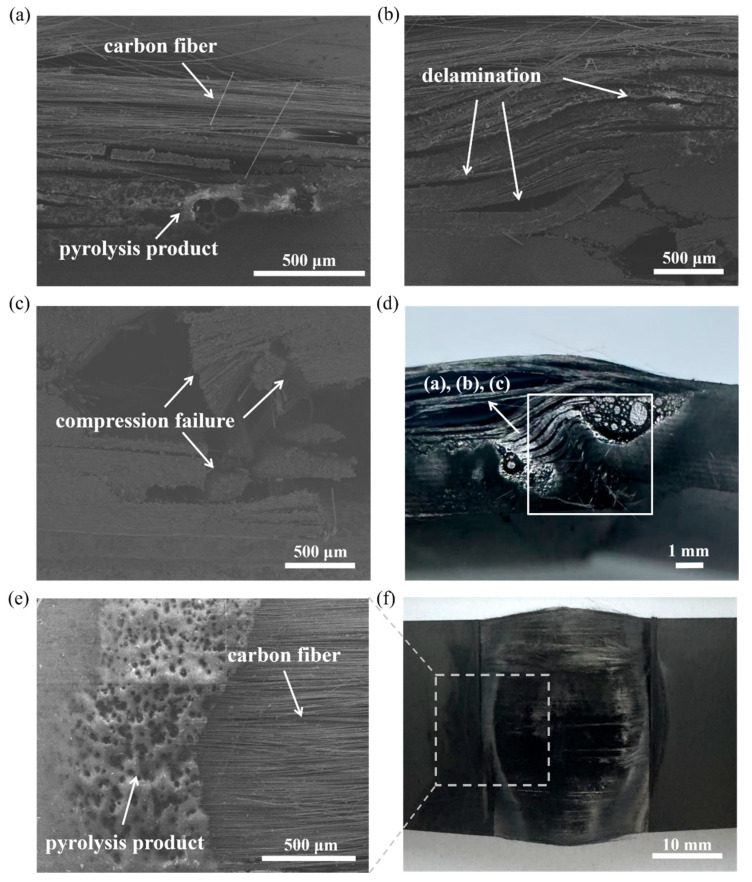
SEM results of the tested CFRP beam specimens, showing (**a**) pyrolysis, (**b**) delamination, (**c**) compression failure, and (**d**) pyrolysis on the top surface on the local region of (**e**,**f**).

Figure 8 illustrates the SEM results of the ablation and failure regions of the CFRP beam. It is apparent that the epoxy resin matrix was ablated/pyrolyzed, and the carbon fibers are exposed and yet still intact, as shown in Figure 8a. These exposed carbon fibers can thus still bear tensile loads, which agrees well with the results in Figure 4. Obvious pyrolysis products and delamination are also shown in Figure 8b,c, respectively. Moreover, the fracture surface of the compressive failure mode on the bottom surface can be observed in Figure 8d.

**Figure 9:**
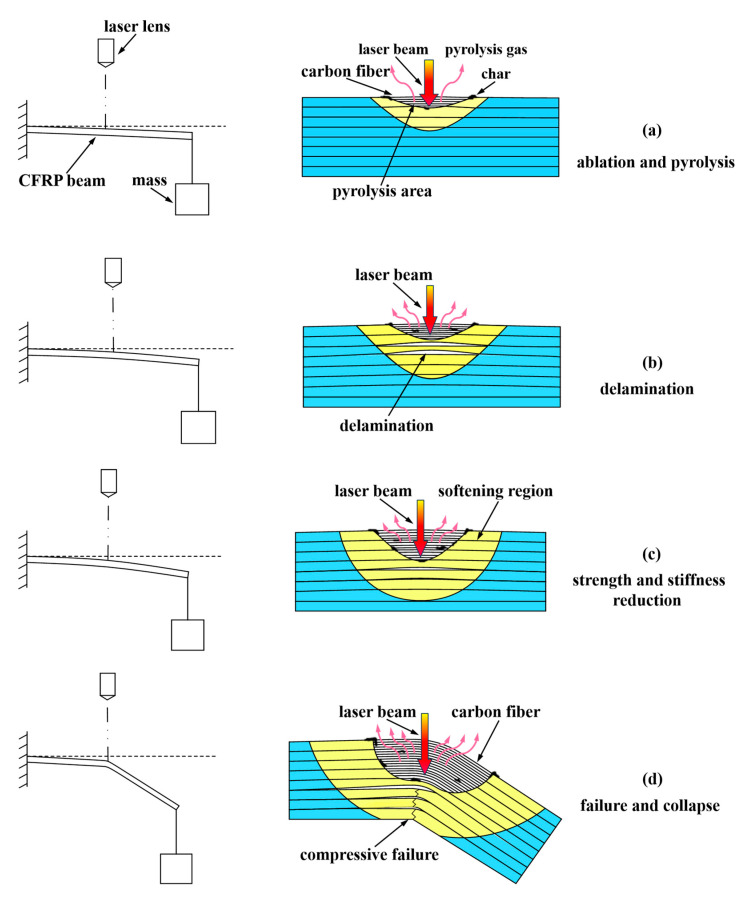
The schematic of the failure mechanism of the pre-stressed CFRP beam under laser ablation: (**a**) ablation and pyrolysis, (**b**) delamination, (**c**) strength and stiffness reduction, and (**d**) failure and collapse.

Combining the ablation experimental results with the TG and SEM results, the deformation and failure mechanism of the pre-stressed CFRP beam subjected to laser ablation has been discussed, and the corresponding schematic is shown in Figure 9. The failure mechanism includes the following four stages: ablation and pyrolysis, delamination, strength and stiffness reduction, and failure and collapse. The details are as follows:

(1) Ablation and pyrolysis. At the initial stage, due to the pre-stress loading, the upper part of the CFRP beam above the neutral axis is in tension, and the lower part below the neutral axis is in compression. When the laser is irradiated on the top surface of the CFRP beam, the temperature increases dramatically and the epoxy resin begins to pyrolyze, and even ablate, as the temperature reaches about 345 °C (pyrolysis point), exposing the carbon fibers. However, the beam remains in tension due to its high pyrolysis temperature, as shown in Figure 9a.
(2) Delamination. Numerous heat fluxes by the laser transfers into the CFRP beam, resulting in an increase in temperature along the thickness direction, which causes the strength between the layers to decrease. When the combined stresses from pre-stress loading, heat expansion, and internal gas induced by pyrolysis exceed the strength between layers, delamination occurs, as shown in Figure 9b.

**Figure 10:**
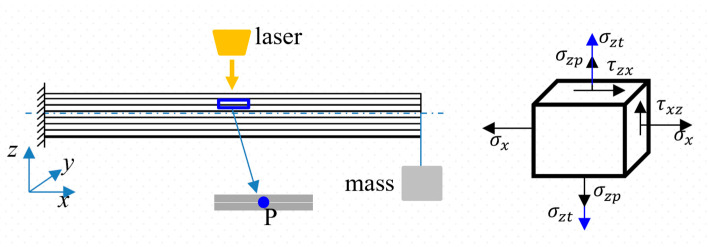
Schematic of the typical stress state in the ablation region.

Figure 10 illustrates the typical stress state in the ablation region.

The normal stress $\sigma_{z}$ is induced by the thermal expansion $\sigma_{zt}$ and pyrolysis gas $\sigma_{zp}$, and the shear stress $\tau_{zx}$ and normal stress $\sigma_{x}\;$ are due to the pre-stress loading, expressed in Formulas (1) and (2). Due to the beam’s complicated stress state, the quadratic stress criterion was employed to discuss the failure mechanism, which is expressed as follows [43]:

(3) $${\left(\frac{\sigma_{n}}{\sigma_{n}^{0}}\right)}^{2}+{\left(\frac{\sigma_{s}}{\sigma_{s}^{0}}\right)}^{2}+{\left(\frac{\sigma_{t}}{\sigma_{t}^{0}}\right)}^{2}=1$$

where $\sigma_{n}$, $\sigma_{s}$, and $\sigma_{t}$ are the normal stress, shear stress in $0^{{}^{\circ}}$ direction, and shear stress in $90^{{}^{\circ}}$ direction, respectively; $\sigma_{n}^{0}$, $\sigma_{t}^{0}$, and $\sigma_{s}^{0}$ are the normal strength, strength in $0^{{}^{\circ}}$ direction, and strength in $90^{{}^{\circ}}$ direction, respectively.

In the present paper, $\sigma_{t}$ is zero. So, Formula (3) can be written as

(4) $${\left(\frac{\sigma_{z}}{\sigma_{z}^{0}}\right)}^{2}+{\left(\frac{\tau_{zx}}{\tau_{zx}^{0}}\right)}^{2}=1$$

where $\sigma_{z}$ and $\tau_{zx}$ are the normal stress and shear stress in $0^{{}^{\circ}}$ direction, respectively; $\sigma_{z}^{0}$ and $\tau_{zx}^{0}$ are the normal strength and strength in $0^{{}^{\circ}}$ direction, respectively.

If $\sigma_{z}$ and $\tau_{zx}$ satisfy Formula (4), damage between the layers would be initiated. And then, based on the Benzeggagh–Kenane (B-K) criterion [44], the delamination between the layers can be analyzed. Delamination was caused by normal stresses $\sigma_{zt}$,$\sigma_{zp}$ and shear stress $\tau_{zx}\;$ between the interface, which belong to the mixed modes of I and II. When the energy is larger than $G_{c}$ [45], delamination occurs.

(3) Strength and Stiffness Reduction. With the growth of the softening area and delamination and pyrolysis zones, the local stiffness and strength of the CFRP beam decrease dramatically, leading to large displacement deformation, as shown in Figure 9c.
(4) Failure and Collapse. When the compression stress in the bottom part of the CFRP beam exceeds the compression strength, the CFRP beam experiences compressive failure and finally collapses, as shown in Figure 9d.

## 4. Conclusions

In the present paper, the thermal–mechanical–chemical coupling response of a pre-stressed CFRP cantilever beam subjected to laser ablation was investigated. Different power densities and pre-stress loading cases were applied, and the corresponding failure times and failure modes of the CFRP cantilever beam were obtained. SEM tests were also conducted to analyze the failure mechanisms. The conclusions are as follows:

1. The laser ablation of the CFRP beam results in the pyrolysis of the epoxy resin, delamination failure, and exposed carbon fibers. The CFRP cantilever beam displayed a compressive failure mode on the bottom surface, which was due to the decrease in strength and stiffness induced by heat softening.
2. Both the power density of the laser and pre-stress loading have an obvious influence on the failure time of the CFRP cantilever beam, whereby the failure time decreases with increasing power density and pre-stress loading. Additionally, the failure time decreased from 19.64 s to 6.52 s with increases in power density (500–1500 W·cm^−2^) and pre-stress loading (300–750 N·cm). Moreover, the results of a two-way ANOVA analysis show that the contribution to failure time by pre-stress loading is 79.8%, which is about 5 times of that by power density. The above results indicate that the pre-stress loading has a greater influence on the failure time of the CFRP beam compared to power density.
3. Based on the experimental results, the failure mechanism of the pre-stressed CFRP cantilever beam is considered to undergo four stages: ablation and pyrolysis, delamination, strength and stiffness reduction, and compressive failure. The CFRP beam ablated due to the high-energy laser, so the matrix pyrolyzed into char, CO_2_, and H_2_O, among others. The carbon fibers were consequently exposed, but they could still bear the tension load, which aligns with that reported in Ref. [42]. Subsequently, the pyrolysis gas, thermal expansion, and pre-stress loading resulted in the occurrence of delamination, which decreased local strength and stiffness of the CFRP beam. Moreover, the heat transfer decreased the local strength and stiffness further. Finally, the CFRP cantilever beam collapsed and failed in compressive mode on the bottom surface when the compressive stress on the bottom surface exceeded the strength that decreased due to the heat transfer.

## Figures and Tables

**Table 1:** Load cases and the corresponding results.

No.	Power Density (W·cm^−2^)	Mass (kg)	Position * (cm)	Pre-Stress Loading (N·cm)	Maximum Temperature ** (°C)	Average Failure Time (s)
1	500	2	15	300	271.9 ± 9.6	19.64 ± 1.31
2	500	5	10	500	247.7 ± 7.5	13.82 ± 0.39
3	500	5	15	750	201.3 ± 19.8	9.44 ± 0.45
4	1000	2	15	300	288.2 ± 6.6	15.78 ± 0.55
5	1000	5	10	500	249.3 ± 12.0	10.44 ± 0.92
6	1000	5	15	750	216.3 ± 6.5	7.18 ± 0.49
7	1500	2	15	300	288.9 ± 6.4	14.58 ± 0.67
8	1500	5	10	500	257.4 ± 5.9	9.56 ± 0.43
9	1500	5	15	750	224.4 ± 10.4	6.52 ± 0.45

* Position: The distance between the center of the laser spot and the free end of the CFRP beam. ** Maximum temperature: The temperature of the position beneath the laser spot center on the bottom surface of the CFRP beam.

**Table 2:** The analysis of two-way ANOVA on the failure time of pre-stressed CFRP beam under laser ablation.

Variation	SS	DF	MS	*p*-Value	Contribution (%)
Power density	78.49	2	39.24	3.1 × 10^−11^	16.7
Pre-stress loading	373.69	2	186.84	4.1 × 10^−17^	79.8
Power density: Pre-stress loading	9.91	4	2.47	7.9 × 10^−4^	2.1
Residual	5.73	18.0	0.31		1.2

SS: sum of squares; DF: degrees of freedom; MS: mean squares; *p*-value: statistical significance.

## Data Availability

The original contributions presented in the study are included in the article, further inquiries can be directed to the corresponding author.
